# Supplementary material for: Knowledge-guided machine learning can improve carbon cycle quantification in agroecosystems
Source: Nat Commun. 2024 Jan 8;15:357. doi: 10.1038/s41467-023-43860-5 (PMC10774286; doi:10.1038/s41467-023-43860-5)
Supplement: Supplementary file 1 — Supplementary Information [file 41467_2023_43860_MOESM1_ESM.pdf]

Supplementary Information for: Knowledge-Guided Machine Learning can improve C cycle quantification in agroecosystems

Supplementary Notes:

Note S1: Training step 1:

The loss function for step 1 with mean-square-error (MSE) based self-paced learning (SPL) method[1,2] can be expressed as:

$$\mathrm{Loss}_{\mathrm{step1}} = \sum_{i=1}^{N} v_{\mathrm{Ra},i} \sum_{t=1}^{\mathrm{Tx}} \frac{(y_{\mathrm{Ra},t,i} - y'_{\mathrm{Ra},t,i})^2}{\mathrm{Tx}N} + \sum_{i=1}^{N} \frac{v_{\mathrm{yield},i}(y_{\mathrm{yield},i} - y'_{\mathrm{yield},i})^2}{N} \quad \text{(S1)}$$

$$v_{\mathrm{Ra},i} = \begin{cases} 1 \text{ when } \sum_{t=1}^{\mathrm{Tx}} \frac{(y_{\mathrm{Ra},t,i} - y'_{\mathrm{Ra},t,i})^2}{\mathrm{Tx}} < \lambda \\ 0 \text{ when } \sum_{t=1}^{\mathrm{Tx}} \frac{(y_{\mathrm{Ra},t,i} - y'_{\mathrm{Ra},t,i})^2}{\mathrm{Tx}} > \lambda \end{cases} \quad \text{(S2)}$$

$$v_{\mathrm{yield},i} = \begin{cases} 1 \text{ when } (y_{\mathrm{yield},i} - y'_{\mathrm{yield},i})^2 > \lambda \\ 0 \text{ when } (y_{\mathrm{yield},i} - y'_{\mathrm{yield},i})^2 < \lambda \end{cases} \quad \text{(S3)}$$

$$\lambda = \lambda_0 \prod_1^{\mathrm{epoch}} K_{\mathrm{growth}} \quad \text{(S4)}$$

where the $\mathrm{Loss}_{\mathrm{step1}}$ is the loss function used in step 1; $y_{\mathrm{Ra},t,i}$ and $y'_{\mathrm{Ra},t,i}$ are the synthetic and predicted Ra flux of the ith sample at time t, respectively; $y_{\mathrm{yield},i}$ and $y'_{\mathrm{yield},i}$ are the synthetic and predicted annual yield of the ith sample, respectively. $v_{\mathrm{Ra},i}$ and $v_{\mathrm{yield},i}$ are the integer multipliers for Ra and yield to decide whether we use the ith sample to calculate the loss; N is the total number of samples for a certain simulation; λ is the threshold with an initial value of λ_0 (0.05) and a growth rate of K_{growth}(1.02). The SPL-MSE method can start training the model with "easier" samples (those with a distance between the synthetic data and the prediction below the threshold λ). As the training epoch increases, the λ will increase, and more "difficult" samples will be involved.

Note S2: Training step 2:

The loss function for step2 can be expressed as:

$$\mathrm{Loss}_{\mathrm{step2}} = \sum_{j=\mathrm{Ra,Rh,NEE}} \sum_{i=1}^{N} \sum_{t=1}^{\mathrm{Tx}} \frac{(y_{j,t,i} - y'_{j,t,i})^2}{\mathrm{Tx}N} + \mathrm{MBLoss}(\mathbf{y'}_{\mathrm{Ra}}, \mathbf{y'}_{\mathrm{Rh}}, \mathbf{y'}_{\mathrm{NEE}}, \mathbf{GPP}) + \mathrm{RspLoss}_{\mathrm{step2}} \quad \text{(S5)}$$

where the first term is the MSE loss for Ra, Rh and NEE predictions; the second term constrains the model predictions with carbon mass balance (GPP - Ra -Rh = -NEE) and can be expressed as:

$$\text{MBLoss}(\mathbf{y'}_{\text{Ra}}, \mathbf{y'}_{\text{Rh}}, \mathbf{y'}_{\text{NEE}}, \mathbf{GPP}) = \sum_{i=1}^{N}\sum_{t=1}^{\text{Tx}} \frac{\text{ReLU}(|\text{GPP}_{t,i} + y'_{\text{Ra},t,i} + y'_{\text{Rh},t,i} + y'_{\text{NEE},t,i}| - \beta_{\text{MB}}|y'_{\text{Ra},t,i} + y'_{\text{Rh},t,i}|)}{\text{Tx}N} \quad (S6)$$

where $\beta_{\text{MB}}|y'_{\text{Ra},t,i} + y'_{\text{Rh},t,i}|$ is the maximum mass balance tolerance that varies with the predicted Ra and Rh, and β_{MB} is the tolerance fraction, set to be 0.01 in this study. Note that the predicted values of Ra and Rh are negative in order to be consistent with a positive direction from the atmosphere to the soil. The third term of Eq. S5 constrains the key response of Rh to TSOC. Ten samples were randomly selected from the synthetic data set to calculate the partial different plot (PDP, Figure S3) by running the model multiple times with adjusted TSOC input and original other input. The predicted Rh from all times and all samples were averaged to one value. The Rh should increase with TSOC increase so that the slope of the PDP ($\text{slope}_{\text{Rh,TSOC}}$) should always be negative. Thus we calculated the response loss by:

$$\text{RspLoss}_{\text{step2}} = \sum_{\text{TSOC=TSOCmin}}^{\text{TSOCmax}} \text{ReLU}(\text{slope}_{\text{Rh,TSOC}}) \quad (S7)$$

where the TSOCmax and TSOCmin are the upper and lower boundary of TSOC in synthetic data, respectively.

Note S3: Training step 3:

320 counties out of the total 637 counties were randomly selected to provide the annual crop yield data during 2000-2020 for the KGML-ag-Carbon model training, while 100 counties were randomly chosen from the rest counties for the model validation. To successfully use the county level NASS data to finetune the KGML-ag-Carbon at field level, we randomly selected 300 points (CDL presented corn/soybean crop fraction bigger than 0.5) in each county to generate a training data set. Then at each epoch, 200 points would be randomly selected from the prepared 300 points at each county to conduct simulations, and then the averaged corn/soybean yield from the points belonging to corn (CDL corn fraction > 0.5) or soybean (CDL soybean fraction > 0.5) would be used to compare with NASS data. The loss for each crop in step 3 can be presented as:

$$\text{Loss}_{\text{step3}} = \sum_{i=1}^{\text{N}_{\text{county}}} \frac{v_{\text{yield},i}(y_{\text{yield},i} - \sum_{j=1}^{\text{N}_{\text{points,i}}} \frac{y'_{\text{yield},i,j}}{N_{\text{points},i}})^2}{N_{\text{county}}} + \text{ThdLoss}_{\text{step3}} + \text{RspLoss}_{\text{step3}} \quad (S8)$$

where the$y_{\text{yield},i}$ is the NASS corn or soybean yield for ith county and $y'_{\text{yield},i,j}$ is the predicted yield for the jth point at the ith county. N_{county} and $N_{\text{points},i}$are the number of counties and points at the ith county, respectively. $\text{ThdLoss}_{\text{step3}}$ is the threshold loss to control yield prediction within the reasonable range, and can be expressed as:

$$\mathrm{ThdLoss_{step3}} = \sum_{i=1}^{N_{\mathrm{county}}} \sum_{j=1}^{N_{\mathrm{points},i}} (\mathrm{ReLU}(0 - y'_{\mathrm{yield},i,j}) + \frac{\mathrm{ReLU}(y'_{\mathrm{yield},i,j} - 0.5 \sum_{t=1}^{\mathrm{Tx}} \mathrm{GPP}_{t,i,j}))}{N_{\mathrm{point},i}} \quad (S9)$$

The $\mathrm{RspLoss_{step3}}$, which is similar to $\mathrm{RspLoss_{step2}}$, is the term to constrain the responses for yield to important drivers including GPP, TSOC, and Year. The $\mathrm{RspLoss_{step3}}$ can be expressed as:

$$\mathrm{RspLoss_{step3}} = \sum_{\mathrm{TSOC=TSOCmin}}^{\mathrm{TSOCmax}} \mathrm{ReLU}(-\mathrm{slope_{yield,TSOC}}) + \sum_{\mathrm{GPP=GPPmin}}^{\mathrm{GPPmax}} \mathrm{ReLU}(-\mathrm{slope_{yield,GPP}}) + \sum_{\mathrm{Year=Yearmin}}^{\mathrm{Yearmax}} \mathrm{ReLU}(-\mathrm{slope_{yield,Year}}) \quad (S10)$$

where $\mathrm{slope_{yield,TSOC}}$, $\mathrm{slope_{yield,GPP}}$ and $\mathrm{slope_{yield,Year}}$ are slopes from PDP representing responses of yield to TSOC, GPP and year, respectively. Those three important factors should always positively affect the yield when increasing if excluding all other influences. Thus we assumed their PDPs should be monotonic increasing over the simulation period (slope > 0). However, finetuning with only NASS data would cause response deviation, especially in out-of-sample scenarios(Figure S5a). Besides, since we averaged yield from multiple points to compare with NASS yield, the point with a relatively larger/smaller yield would have a larger gradient for parameter learning than with a similar yield, which would further exacerbate the deviation of responses (Figure S5b). Finetuning with NASS data together with synthetic data and physical losses (Eq. S9-10), the responses can be controlled in a reasonable range and thus can benefit out-of-sample predictions (Figure S5c).

Note S4: Training step 5:

The 11 sites were shuffled in each epoch before inputting into the model for training and validation. The loss was calculated based on results from 11 sites and could be expressed as:

$$\mathrm{Loss_{step5}} = \sum_{i=1}^{N_{\mathrm{site}}} \Bigg(\frac{\sum_{t=1}^{\mathrm{Tx}} \left(y_{\mathrm{NEE},t,i} - y'_{\mathrm{NEE},t,i}\right)^2}{\mathrm{Tx}} + \frac{\sum_{\mathrm{Tx1}<t<=\mathrm{Tx2}} \left(y_{\mathrm{Reco},t,i} - y'_{\mathrm{Ra},t,i} - y'_{\mathrm{Rh},t,i}\right)^2}{\mathrm{Tx2} - \mathrm{Tx1}} + \sum_{0<t<=\mathrm{Tx1},\mathrm{Tx2}<t<=\mathrm{Tx}} \frac{(y_{\mathrm{Reco},t,i} - y'_{\mathrm{Rh},t,i})^2}{\mathrm{Tx1} + \mathrm{Tx} - \mathrm{Tx2}} \Bigg) / N_{\mathrm{site}} + \mathrm{MBLoss}\left(\mathbf{y}'_{\mathrm{Ra}}, \mathbf{y}'_{\mathrm{Rh}}, \mathbf{y}'_{\mathrm{NEE}}, \mathbf{GPP}\right) + \mathrm{RspLoss_{step5}} \quad (S11)$$

where the first term is MSE loss for NEE and Reco. Tx1 and Tx2 are the time before all planting begins for corn and soybean, and the time after all harvest activities, respectively (for this study, Tx1=105 DOY and Tx2 = 300 DOY). We assumed that before planting and after harvest, Rh from the soil would be the dominant component for Reco. Thus we used predicted Rh and observed Reco during that period to calculate the MSE loss. The N_{site} is the total number of sites. The second term is the same mass balance equation as equation 17. The third term is the response loss to maintaining enough prior biophysical/chemical knowledge learned from pretraining by constraining the responses of Rh and Ra to all input features not varying too much from the responses after step 4. The $\mathrm{RspLoss}_{\mathrm{step5}}$ can be expressed as:

$$\mathrm{RspLoss}_{\mathrm{step5}} = \sum_{j=\mathrm{Ra,Rh}} \sum_{f=1}^{N_{\mathrm{feature}}} \mathrm{ReLU}(r_{j,f,\min} - r_{j,f}) \qquad (S12)$$

where the $r_{j,f}$ is the Pearson correlation coefficient between the PDPs from step 4 (pretraining) and step 5 (finetuning) for responses of the variable j to the fth feature, representing the similarity of two responses before and after finetuning. The $r_{j,f,\min}$ is the tolerance minimum similarity for responses of the variable j to the fth feature ($r_{j,f,\min}$ is set to 0.8 for all daily dynamics features and 0.9 for other static features). This loss term can keep the reasonable responses from detouring during finetuning and maintain enough prior knowledge, even with many training epochs and little observed data (Figure S7).

It should be noted that our training configuration may not be a totally clean setup for data leakage, as the inputs for pretraining (2000-2018 in 3I states) and fine-tuning (2000-2020 in the US Midwest including 12 states) may share similar climate input information in three states. However, we've taken meticulous steps to mitigate the impact of input leakage to the utmost extent on robustness analysis (as illustrated in Fig. 2), by employing distinctly different outputs (synthetic data vs. observations) and utilizing input data at varying scales (county-level vs. pixel-level). Furthermore, to confirm the robustness of our KGML-ag-Carbon model, we have examined its performance in full factorial tests, as depicted in Fig. S11. Our findings demonstrate that even compared to a pure ML model with pretraining (indicated in red), the KGML-ag-Carbon model (depicted in light brown) maintains superior performance in all scenarios.

Supplementary Discussion:

We implemented a mass balance approach to estimate ΔSOC from KGML-ag-Carbon estimated NEE and crop yield. Generally, Fig. S14a indicates relatively stronger correlations between NEE and input variables, compared with yield. Therefore, the spatial patterns of ΔSOC are likely shaped more by NEE. Since we define the positive direction of NEE as from the soil to the atmosphere, a negative correlation coefficient signifies a decrease in atmospheric carbon but may also indicate an increase in soil carbon. We have further decomposed the input factors into two categories: climate factors and soil factors, to better explain the spatial pattern of ΔSOC.

Climate factors influence the ΔSOC mainly through influencing the GPP (Fig. S14, Fig. 5). The GPP's heatmap patterns closely mirror that of ΔSOC in Fig. S14a. Regions in the southern

areas tend to experience relatively higher radiation, temperature, precipitation, and humidity, especially when compared to their northern counterparts. As observed in Zhou et al. (2021)[3], when air temperature (strongly correlated with radiation as presented in Fig. S14b) is less than 30℃, GPP increases quickly along with the temperature increase. Meanwhile, the air humidity (strongly correlated with precipitation as presented in Fig. S14b) influences GPP through affecting crop stomatal conductance and the increase of humidity (when below 10hPa) will increase the GPP accordingly. Consequently, the southern regions exhibit higher GPP, contributing to an increased influx of carbon input into the soil (crop residue in Fig. S14a). This phenomenon potentially results in higher ΔSOC values in these areas. Furthermore, our observations indicate a strong positive correlation between Ra and specific climate parameters (i.e. radiation, temperature, humidity, and precipitation). However, it's crucial to note that the total Reco may not exhibit a correspondingly strong response (Fig. S14a). This could partly be attributed to the negative correlation observed between Rh and certain climate factors. This negative relationship may stem from the shared patterns between climate factors and soil properties such as TSOC, TFC, and TWP (relatively higher in the north and lower in the south). Nonetheless, it's important to acknowledge that these discrepancies could also arise from the partitioning of Reco into Ra and Rh components. The KGML-ag-Carbon, which was constrained using Reco, necessitates further validation and examination using the partitioned data of Ra and Rh to elucidate these correlations more definitively.

Soil factors influence the ΔSOC primarily by affecting Rh. We find that Rh exhibits heatmap patterns that are nearly the inverse of that of ΔSOC (Fig. S14a). Higher levels of TSOC tend to correlate with optimal soil conditions characterized by increased substrate availability for microbes and improved drainage (e.g. higher TFC and TWP). These favorable conditions then drive up Rh rates, leading to reduced NEE in the soil and ultimately contributing to a decrease in ΔSOC (Zhou et al. 2023)[4]. The northern regions exhibit higher levels of TSOC, TFC, and TWP when compared to their southern counterparts. Consequently, these regions often experience a net loss of carbon.

It is important to note that the estimated SOC increase in the southern US Midwest may be subject to overestimation. This overestimation is primarily attributed to the relatively lower corn and soybean fractions (<60%; as seen in Fig. S1 and Fig. S13a). These lower fractions contribute to more pronounced mixed pixel effects when remotely sensed GPP is used as an input. Fig. S16a-b illustrates the issue further, showing that within a 250-meter pixel, various land covers can coexist, leading to a mixed GPP signal. If vegetation types with higher GPP (e.g., forests) than the identified crop types are present in a single pixel, it can result in an overestimate of remotely sensed GPP, subsequently leading to an overestimated increase in SOC. To mitigate this effect, we have excluded regions where the total corn and soybean fractions are below 0.5 (Fig. S13a; Fig. 5b). Furthermore, the absence of EC flux tower data in the southern region (indicated by blue stars in Fig. S1) may limit the performance of our model. While KGML-ag-Carbon has demonstrated better performance in data-scarce scenarios compared to traditional PB and pure ML models (Fig. 2; Fig. S9), incorporating new data could enhance the reliability of our estimates. For instance,

Fig. 2d highlights that constraining NEE with one site of flux tower data can improve the out-of-sample prediction accuracy, with the mean R-squared value improved from 0.91 (without data constraints) to 0.93. Moreover, our approach employs a mass balance method to estimate ΔSOC, which includes crop residues that may not be converted into SOC within the specified period. So far, there is no available data to effectively exclude crop residues from the estimation. Specifically, ΔSOC = -NEE - Yield = turnovered SOC (humus) + undecomposed crop residue. Unfortunately, there is a lack of comprehensive and large-scale data to effectively distinguish between turnovered SOC and the remaining crop residue. Using a multi-year average for ΔSOC estimation with the assumption that no residue will be removed, the term undecomposed crop residue will be minimized.

In summary, the spatial patterns of ΔSOC are mainly governed by regional climate and soil factors. The relatively warmer, wetter climate in the south, coupled with lower TSOC, predominantly contributes to carbon gain. Conversely, the northern regions, characterized by colder, drier conditions and higher TSOC levels, are primarily responsible for carbon loss. We conducted multivariate linear regression analyses to further examine the total influence of climate or soil factors on ΔSOC. The R-squared values were 0.11 ($p < 0.0001$) and 0.43 ($p < 0.0001$) for climate and soil factors, respectively, indicating that the soil may play a relatively more dominant role in explaining the spatial pattern of ΔSOC. It's worth noting that this study doesn't delve into the influence of management practices, given its historical simulation approach employing predefined crop types and rotations. Evaluating various management practices would necessitate substantial research efforts and remains a prospective avenue for future investigations.

Supplementary Figures:

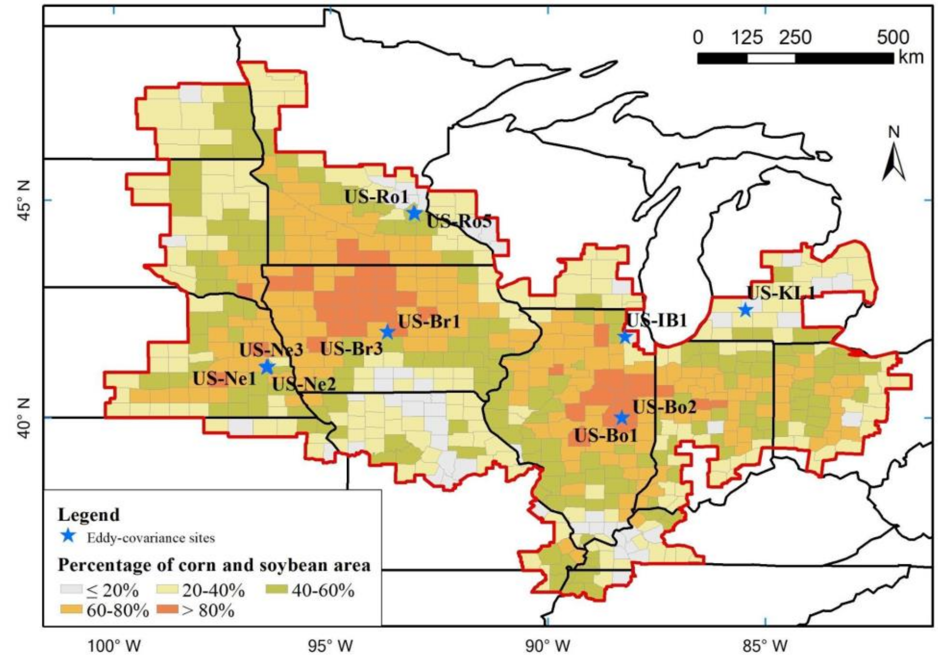

Figure S1. Study region (red lines highlighted region) and the location of agroecosystem eddy-covariance sites used for the KGML-ag-Carbon model finetune and validation.

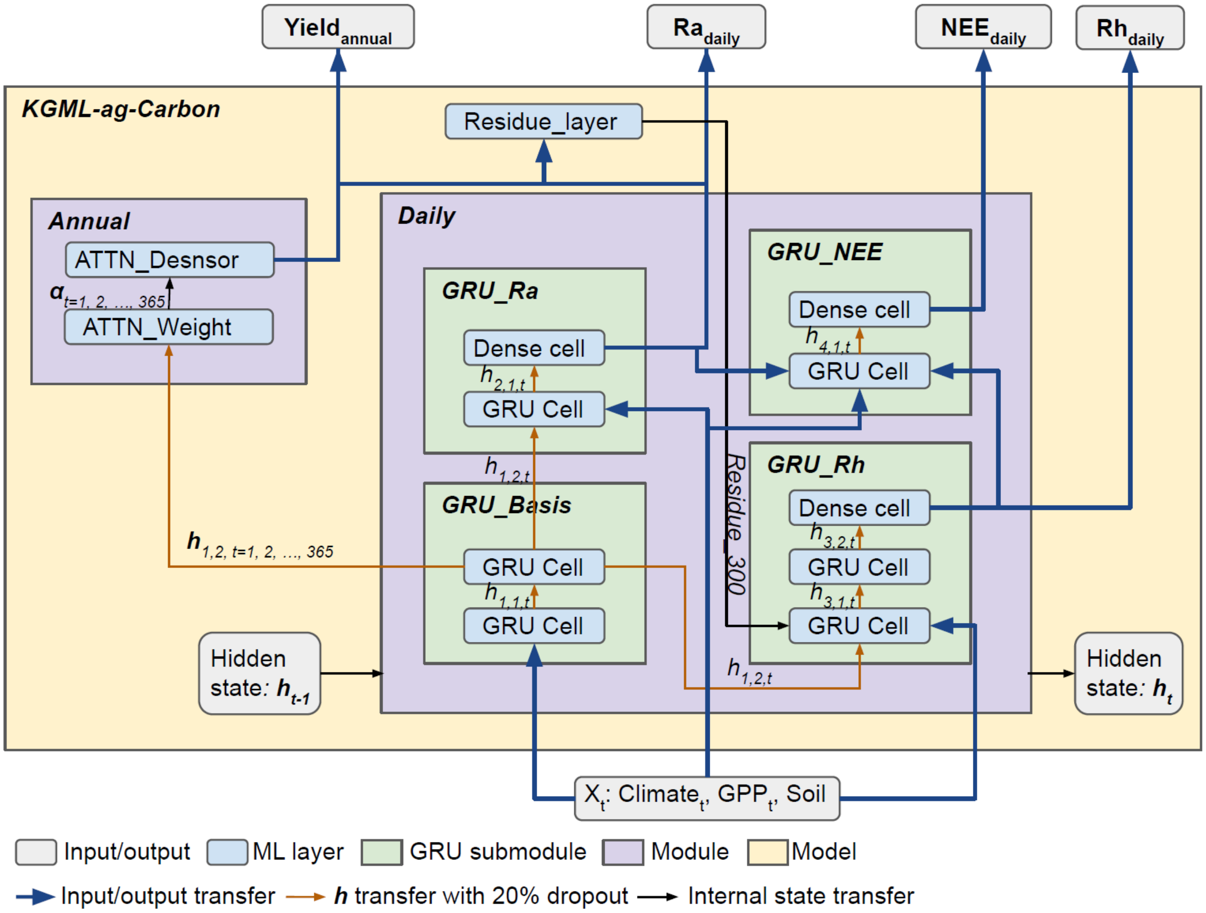

Figure S2. The basic structure of the KGML-ag-Carbon model developed in this study.

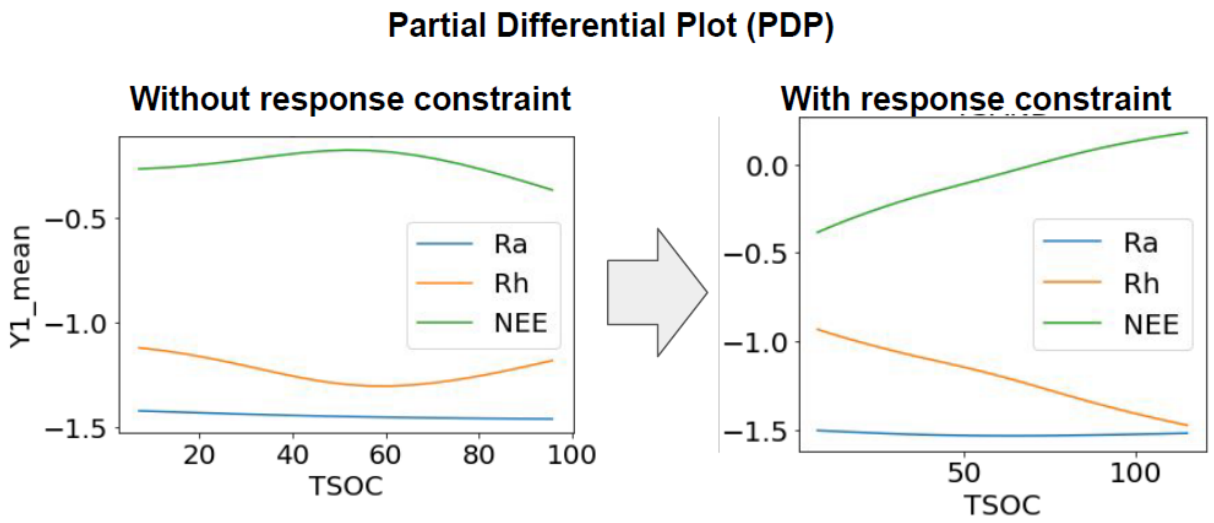

Figure S3.Illustration of using a partial difference plot (PDP) to control the model-predicted Ra, Rh, and NEE responses to soil organic carbon input.

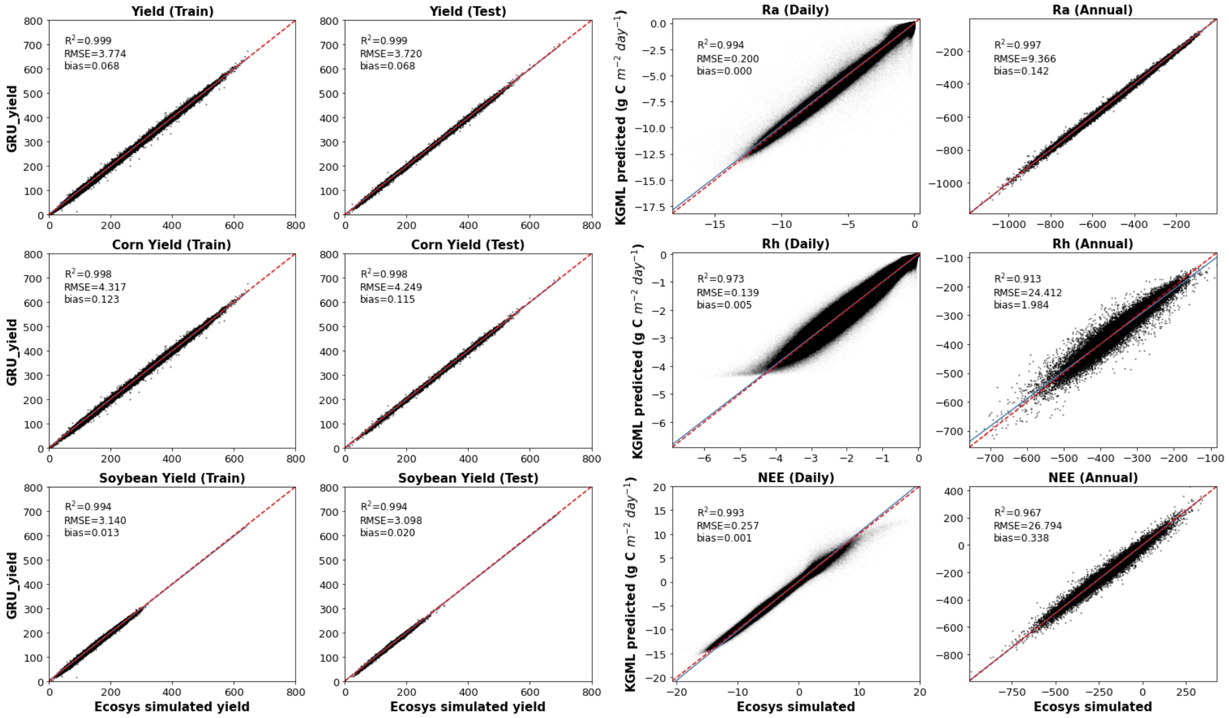

Figure S4. The performance of KGML-ag-Carbon in Yield, Ra, Rh, and NEE prediction based on *ecosys* generated synthetic data. Source data are provided as a Source Data file.

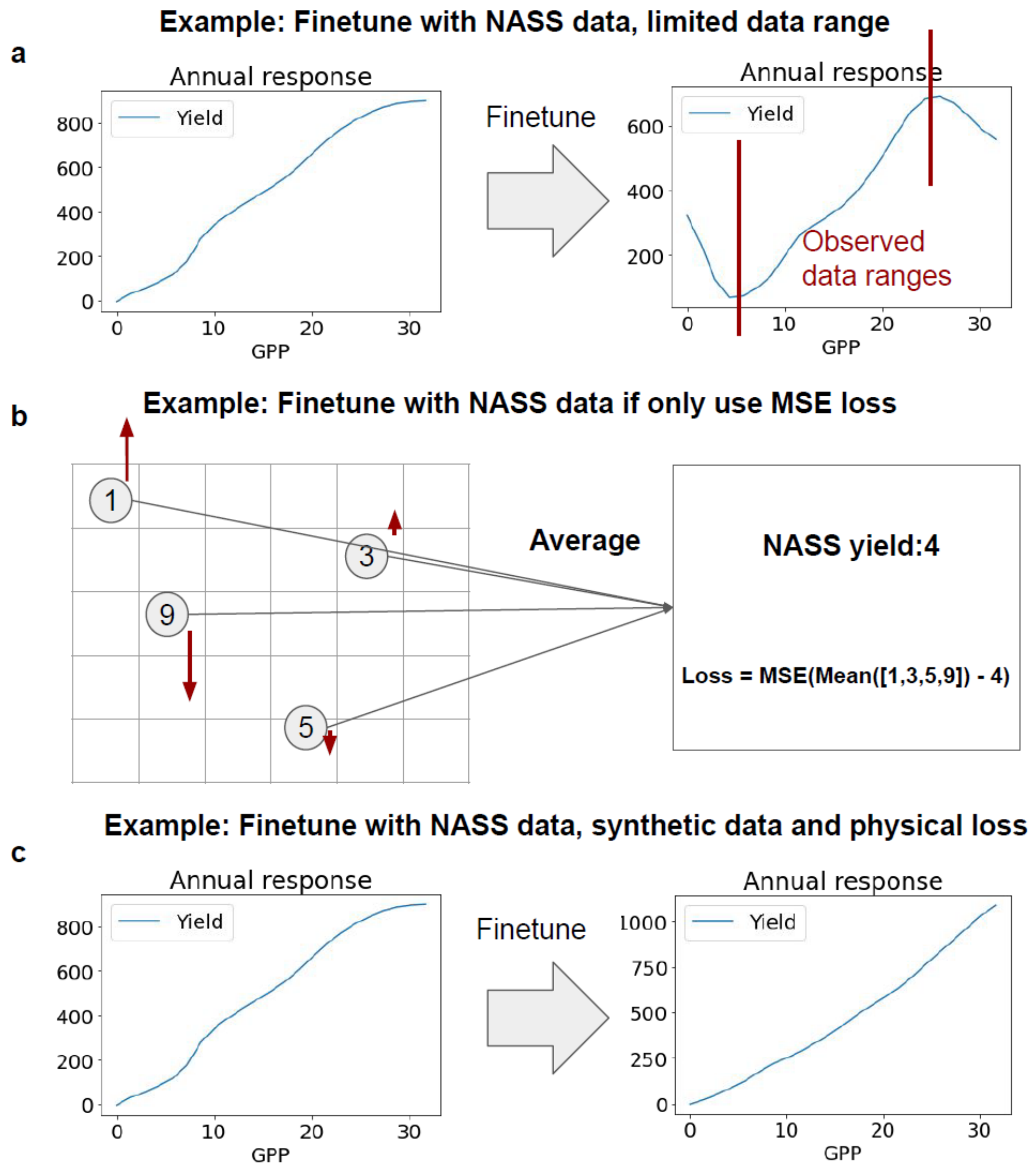

Figure S5. Examples of limited data range influences on finetuning (a), challenges using coarse data to constrain model (b), and finetune results with the method presented in our study (c).

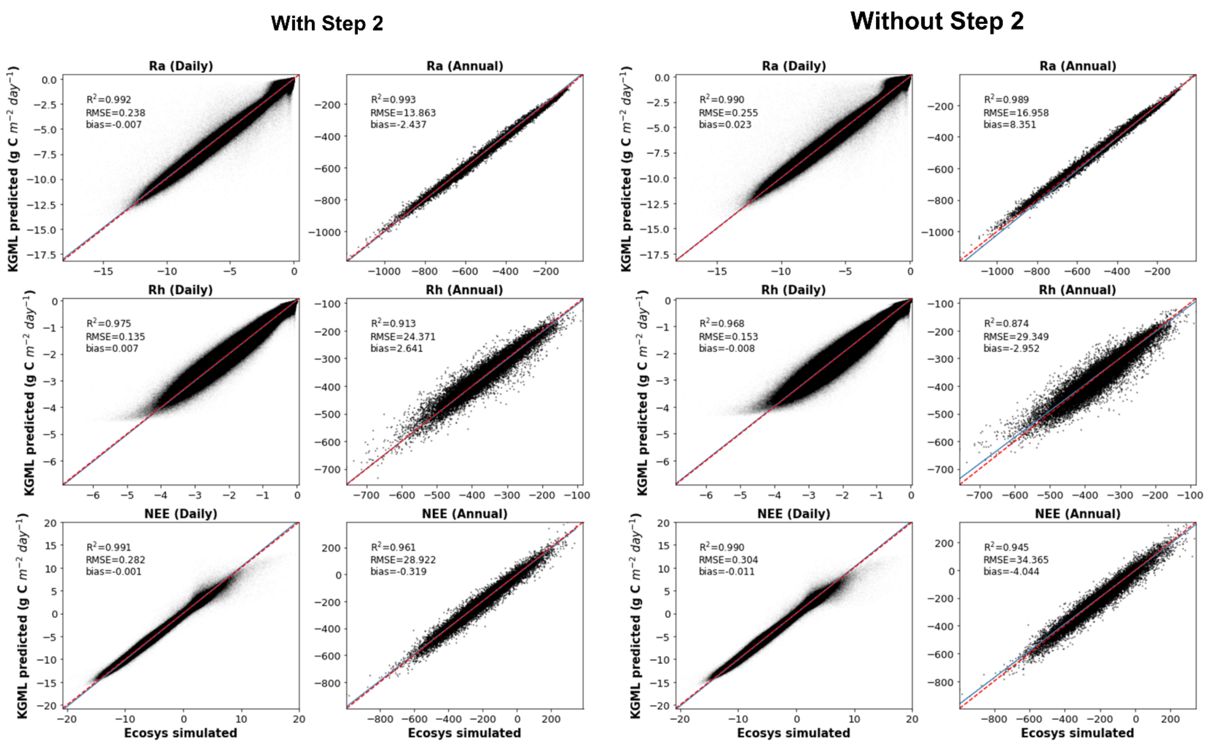

Figure S6. Comparisons of the performance in Ra, Rh, and NEE prediction after step 4 pretraining between KGML-ag-Carbon models with step 2 (left panel) and without step2 (right panel). Source data are provided as a Source Data file.

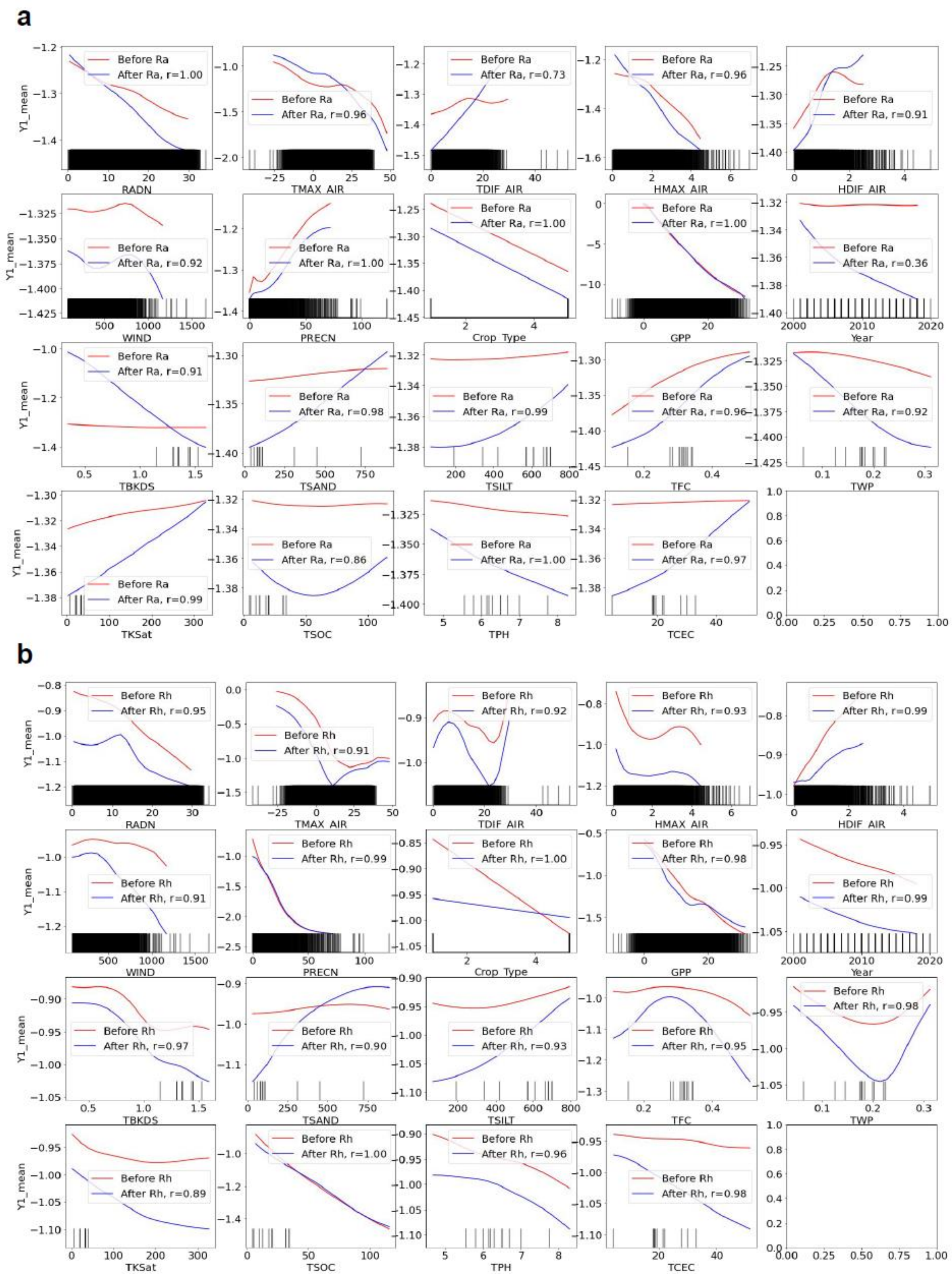

Figure S7. Comparisons of responses of Ra and Rh to all input features between the KGML-ag-Carbon models before step 5 finetuning (red) and after (blue). Each vertical black line represents a data point used for finetuning. Source data are provided as a Source Data file.

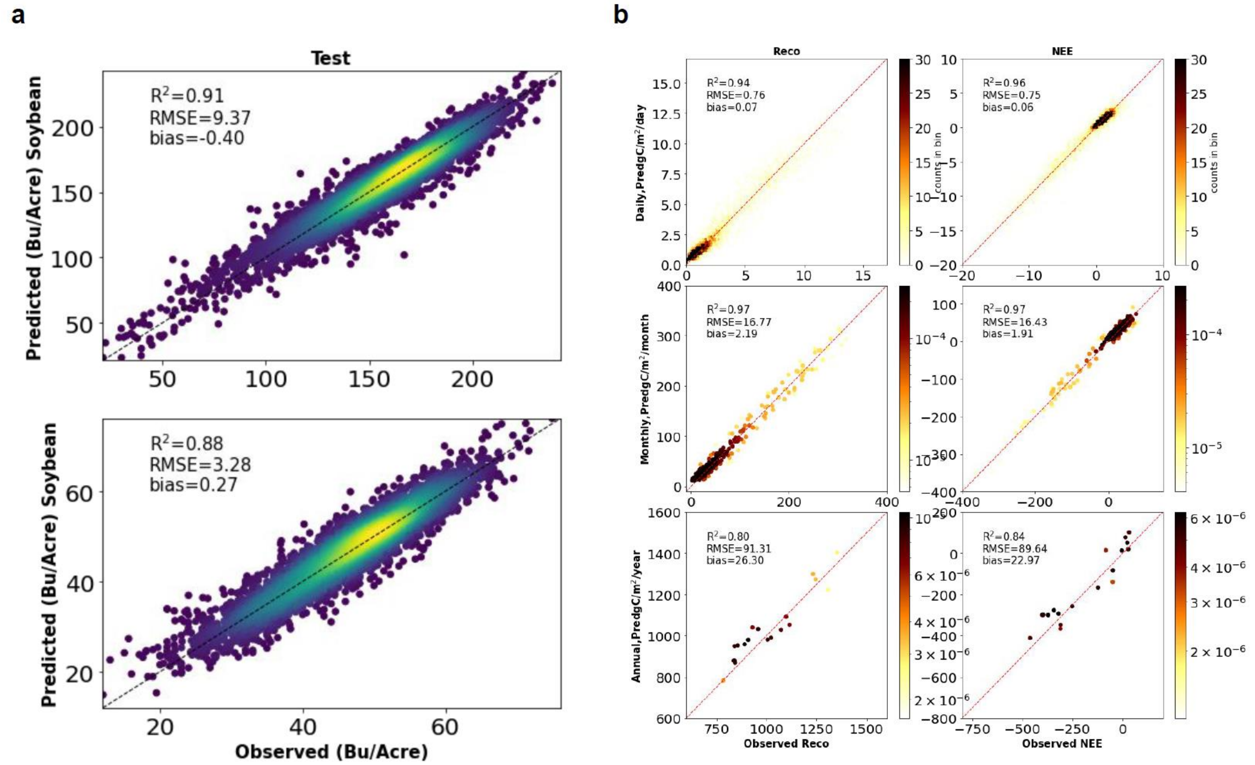

Figure S8. Out-of-sample performance in crop yield estimation and Ra, Rh, and NEE prediction after fine-tuning. Source data are provided as a Source Data file.

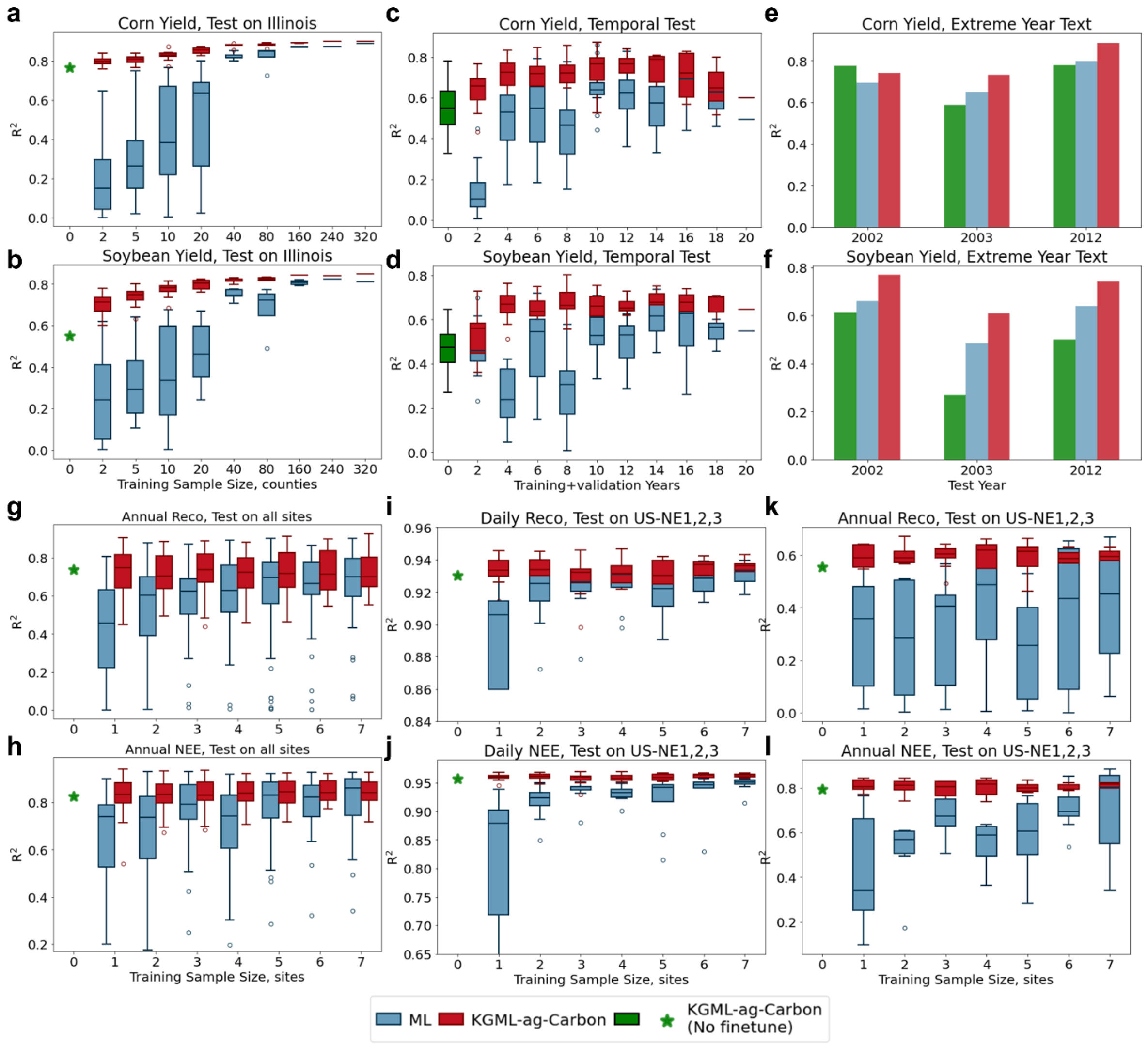

Figure S9. Robust test results besides Figure 2 to compare pure ML model (blue), KGML-ag-Carbon model before fine-tuning (green), and KGML-ag-Carbon model (red), including (a-b) Yield tested on Illinois. n = 1, 50, 20, 10, 10, 5, 4, 2, 1, and 1 independent experiments for model ensembles with a training sample size of 0, 2, 5, 10, 20, 40, 80, 160, 240, and 320, respectively; (c-d) Yield trained using NASS data for a specified time period (given in x-axis) and tested on the remaining period. n = 21, 19, 17, 15, 13, 11, 9, 7, 5, 3, and 1 different years for checking model's performance training with 0, 2, 4, 6, 8, 10, 12, 14, 16, 18, and 20 years of data; (e-f) Yield tested on extreme years; (g-h) Annual flux tested on all sites. n = 8 independent experiments for model ensembles; (i-j) Daily flux tested on sites US-NE1, 2 and 3. n = 8 independent experiments for model ensembles; and (k-i) Annual flux tested on sites US-NE1, 2 and 3. n = 48 independent experiments for model ensembles. A cross-validation method together with ensemble methods are used for robustness tests for fluxes using 11 flux tower sites (g-l). Each box plot illustrates the first and third quartiles (lower and upper box edges), median (central line), and minimum and maximum (lower and upper whiskers), with outliers as round circles. Source data are provided as a Source Data file.

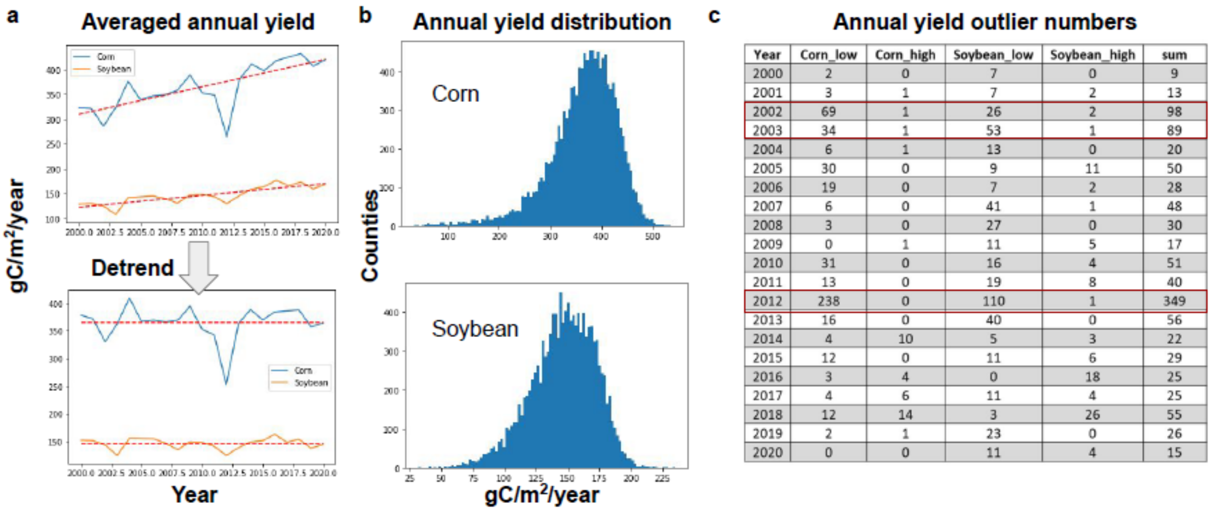

Year	Corn_low	Corn_high	Soybean_low	Soybean_high	sum
2000	2	0	7	0	9
2001	3	1	7	2	13
2002	69	1	26	2	98
2003	34	1	53	1	89
2004	6	1	13	0	20
2005	30	0	9	11	50
2006	19	0	7	2	28
2007	6	0	41	1	48
2008	3	0	27	0	30
2009	0	1	11	5	17
2010	31	0	16	4	51
2011	13	0	19	8	40
2012	238	0	110	1	349
2013	16	0	40	0	56
2014	4	10	5	3	22
2015	12	0	11	6	29
2016	3	4	0	18	25
2017	4	6	11	4	25
2018	12	14	3	26	55
2019	2	1	23	0	26
2020	0	0	11	4	15

Figure S10. Extreme year selecting method. The extreme years were selected by detecting the outlier numbers (not in the range of mean ± two times the STD) for each year based on a yield distribution calculated from the detrended yield for all counties and all years.

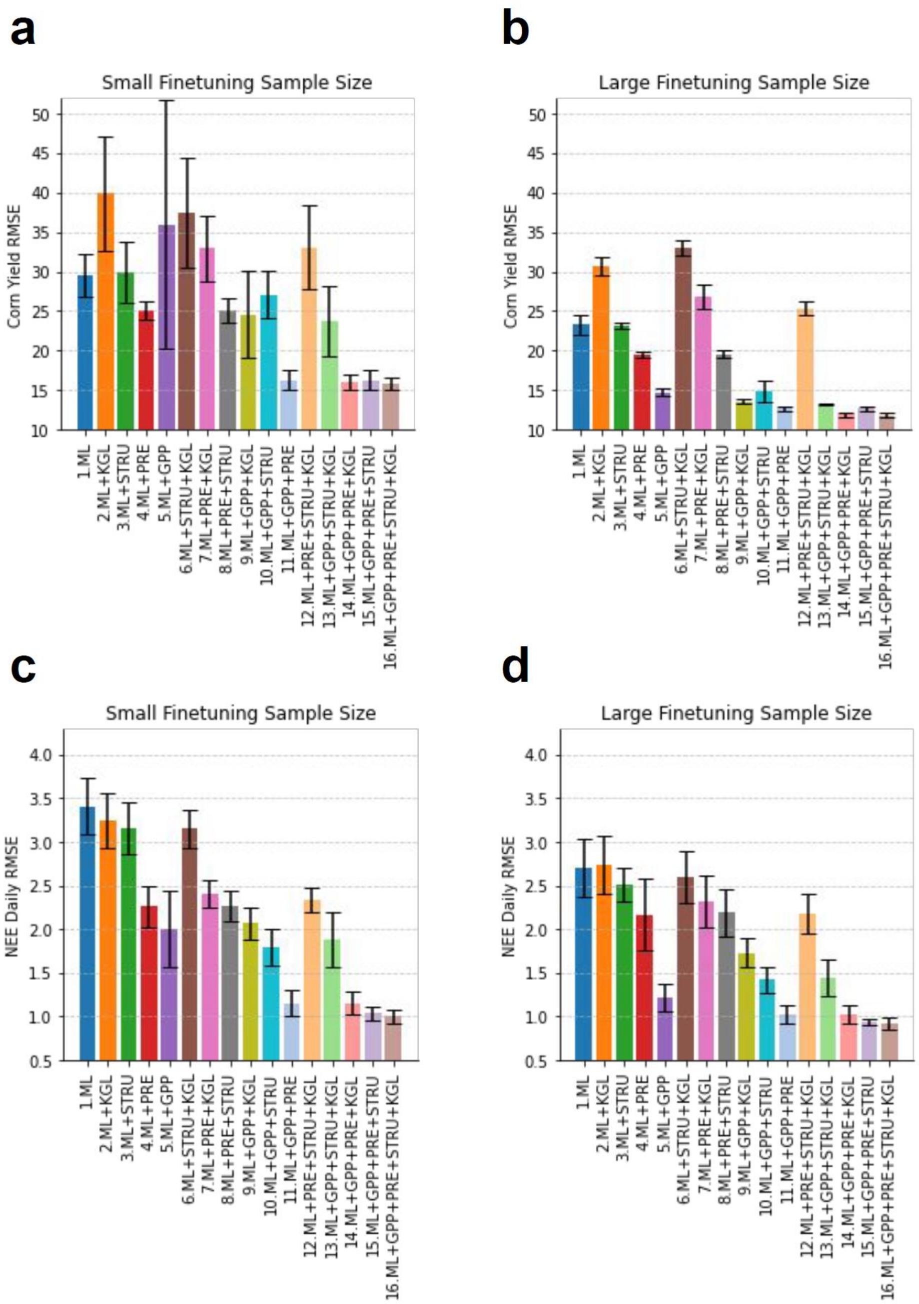

Figure S11. Full-factorial tests for the KGML-ag-Carbon. (a-b) presented impacts of different components of the KGML-ag-Carbon model on the annual corn yield prediction accuracy, when the testing models trained with 5 or 40 counties out of 637 counties, and n = 20 and 5 independent experiments, respectively; and (c-d) presented impacts on the daily NEE flux prediction accuracy when trained with 1 or 7 sites out of 11 sites, and n = 6 independent experiments. Data in each bar plot is presented as mean values +/- standard deviation. ML represents machine learning, PRE represents pretraining using synthetic data, KGL represents the knowledge-guided loss, and STRU represents the knowledge-guided structure. Source data are provided as a Source Data file.

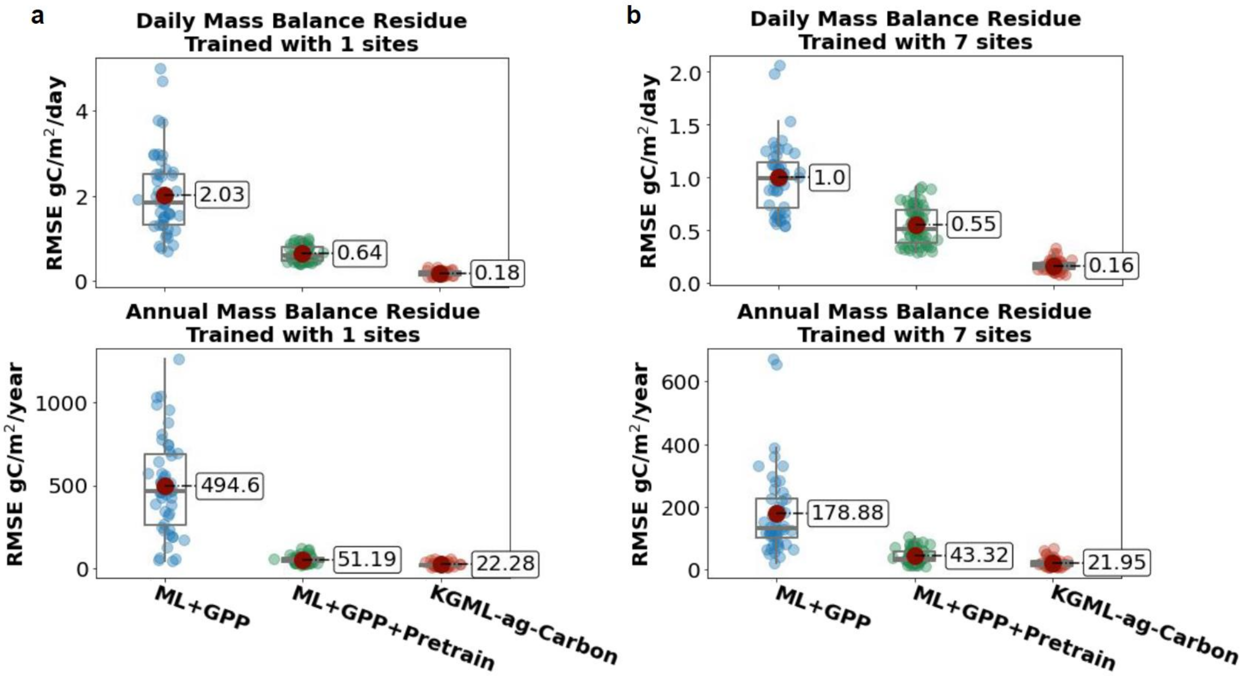

Figure S12. The knowledge-guided components (Pretrain, structure, and KG loss function) influence on reducing mass balance residues at daily and annual scales when the model trained with (a) 1 site and (b) 7 sites out of 11 flux tower sites. Source data are provided as a Source Data file.

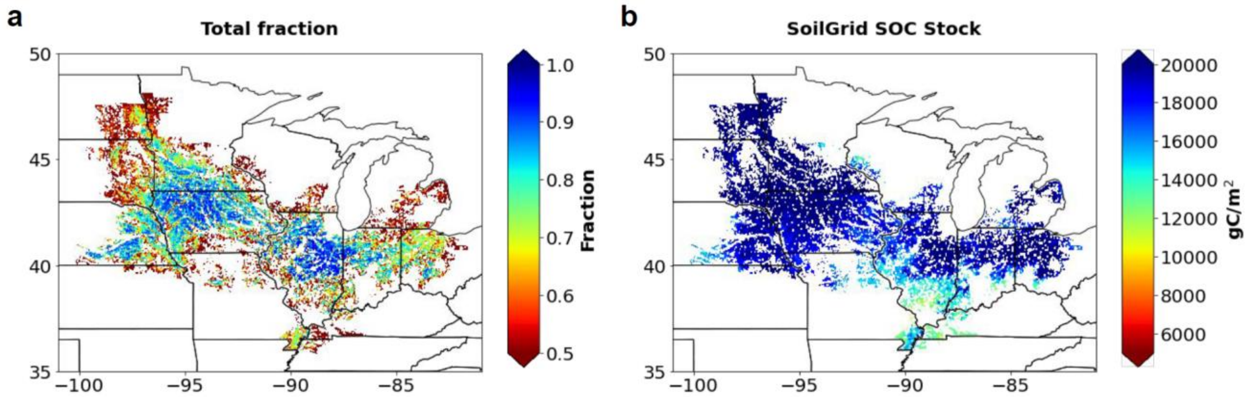

Figure S13. The spatial distributions of (a) the total fraction of areas planting corn or soybean in each pixel, derived from CDL and averaged for period of 2000-2020; and (b) the SOC stock derived from SoilGrid organic carbon density (OCD) in each layer within 0-200 cm depth of soil (representing the whole soil profile).

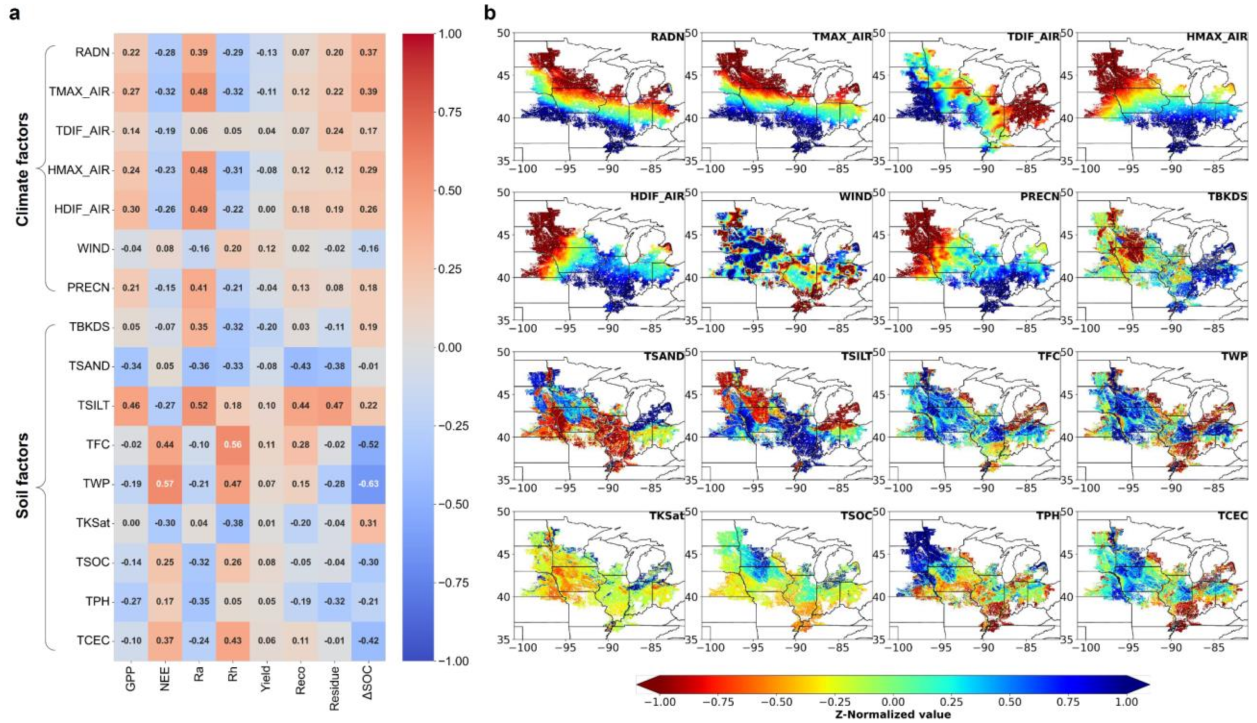

Figure S14. The assessments of correlations between KGML-ag-Carbon input variables and GPP, NEE, Ra, Rh, Yield, and ΔSOC. (a) Pearson's correlation coefficients between each pair of variables. All p values < 0.0001 due to the huge amount of samples (~4 million); and (b) Spatial distributions of input variables. The region is selected based on the total fraction of areas planting corn or soybean over 0.5 which is presented in Figure S13a. The GPP is an input for KGML-ag-Carbon. NEE, Ra, Rh, and Yield are output from KGML-ag-Carbon. ΔSOC is estimated with the mass balance approach using KGML-ag-Carbon estimated NEE and Yield. The positive direction of NEE is from soil to atmosphere. Source data are provided as a Source Data file.

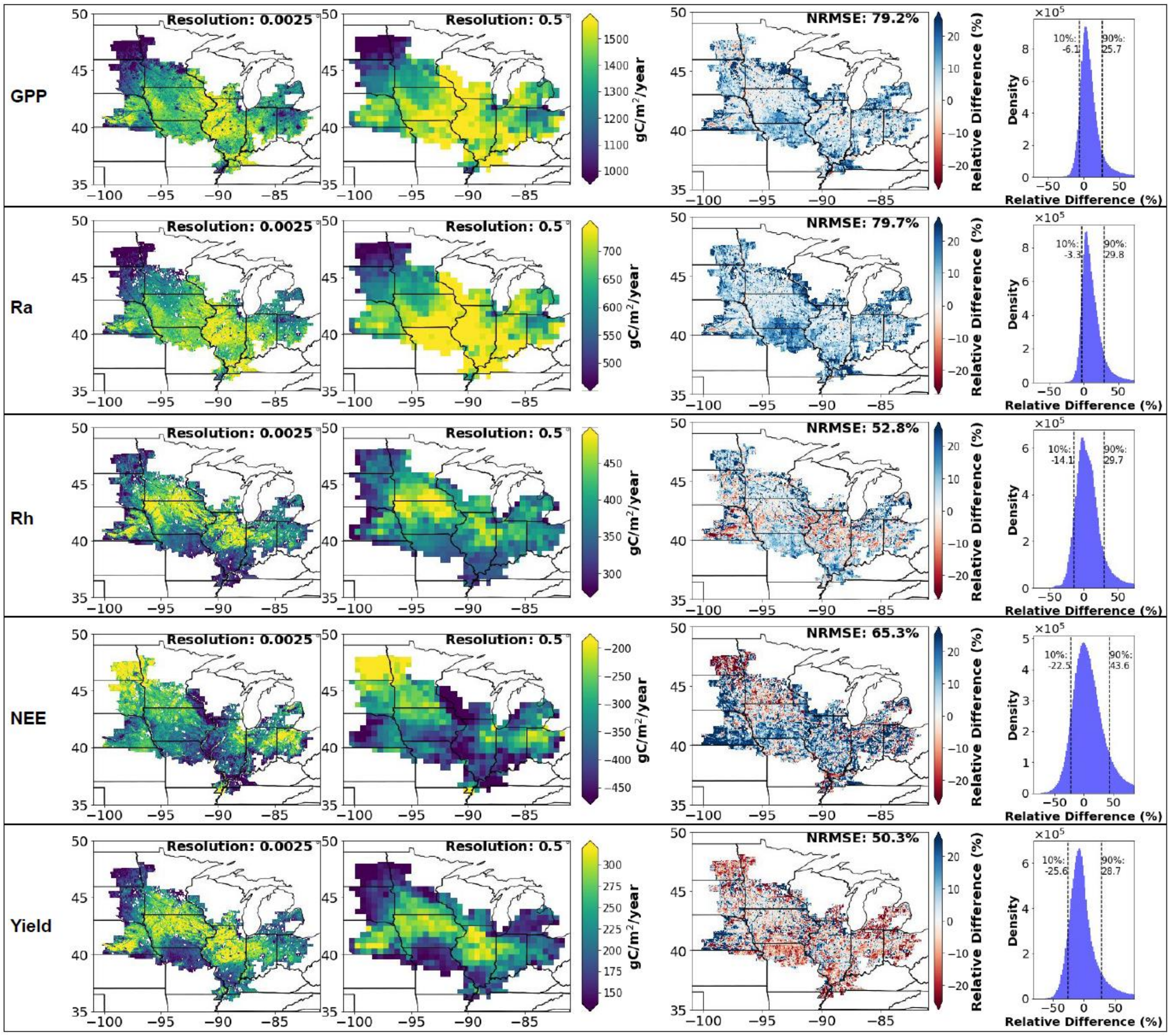

Figure S15. The impacts of coarse resolution on KGML-ag-Carbon estimated (a) GPP, (b) Ra, (c) Rh, (d) NEE, and (e) Yield. Within each panel, 0.0025-degree-resolution estimation, the 0.5-degree-resolution estimation, the difference between coarse-resolution (0.5 degrees) and fine-resolution (0.0025 resolution) estimations relative to the fine-resolution estimation, and the histogram distribution of the relative differences are presented sequentially. Source data are provided as a Source Data file.

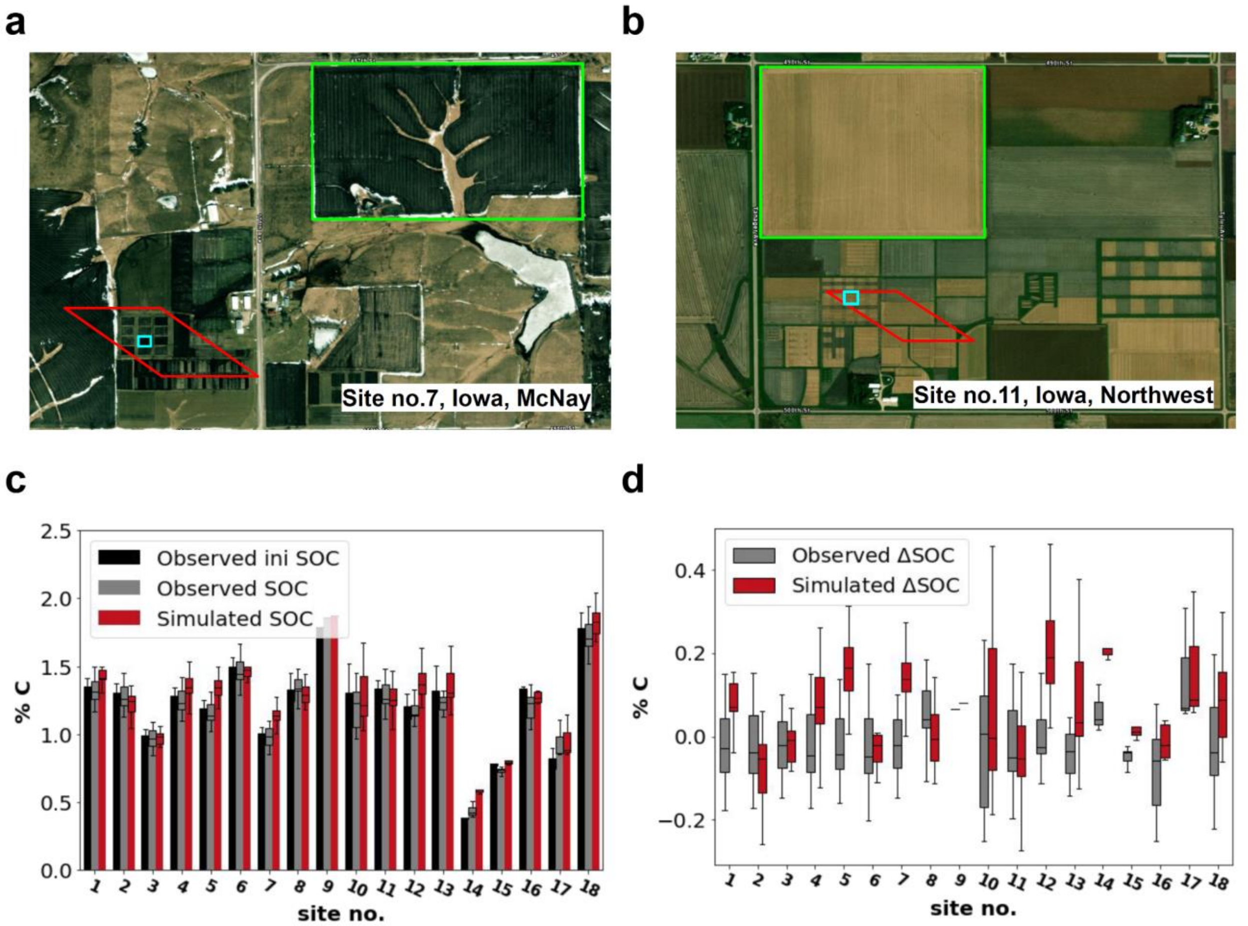

Figure S16. Illustration of scale mismatch between ΔSOC measurements and model estimates (a-b) and validation of KGML-ag-Carbon estimates of ΔSOC (c-d) and. Site details can be found in Table R1. In panels (a-b), the blue boxes indicate the plot measurement areas, the red boxes indicate the KGML-ag-Carbon estimate areas that encompass the plot measurement areas, and the green boxes indicate real agricultural fields in the US Midwest. In panels (c-d), box plots depict the distributions of observations or simulations from different plots. n = 15, 15, 15, 15, 15, 15, 15, 6, 1, 10, 14, 14, 14, 3, 9, 6, 3, 6 independent experimental plots for 1-18 sites, respectively. Basemaps in (a-b): ESRI. World_Imagery_Firefly [ArcGIS Pro Map Service]. Scale not given. Accessed November 17, 2023. Available from: https://www.arcgis.com/home/item.html?id=a66bfb7dd3b14228bf7ba42b138fe2ea. Source data are provided as a Source Data file.

Table S1. Sites used for validating SOC estimations using KGML-ag-Carbon.

Site no.	State	Location	Latitude	Longitude	Length	Plots N	Period[a]	Reference[b]
1	Iowa	Kanawha	42.9368	-93.7987	13	15	2002-2014	Al-Kaisi, M. M., and Kwaw-Mensah, D., 2020
2	Iowa	Sutherland	42.9304	-95.5273	13	15	2002-2014	Al-Kaisi, M. M., and Kwaw-Mensah, D., 2020
3	Iowa	Nashua	42.9318	-92.5727	13	15	2002-2014	Al-Kaisi, M. M., and Kwaw-Mensah, D., 2020
4	Iowa	Armstrong	41.3052	-95.1732	13	15	2002-2014	Al-Kaisi, M. M., and Kwaw-Mensah, D., 2020
5	Iowa	Ames	41.9928	-93.6555	13	15	2002-2014	Al-Kaisi, M. M., and Kwaw-Mensah, D., 2020
6	Iowa	Crawfordsville	41.2171	-91.5086	13	15	2002-2014	Al-Kaisi, M. M., and Kwaw-Mensah, D., 2020
7	Iowa	McNay	40.9725	-93.4263	13	15	2002-2014	Al-Kaisi, M. M., and Kwaw-Mensah, D., 2020
8	Minnesota	Rosemount	44.7508	-93.0746	6	18	2000-2005	Venterea, R. T., et al., 2006
9	Iowa	Boone	42.0070	-93.7873	8	2	2008-2015	Ibrahim, M. A., et al., 2018
10	Iowa	Central	42.0167	-93.7833	15	10	2000-2014	Poffenbarger, H. J., et al., 2017
11	Iowa	Northwest	42.9292	-95.5403	15	14	2000-2014	Poffenbarger, H. J., et al., 2017
12	Iowa	South	40.9667	-93.4167	15	14	2000-2014	Poffenbarger, H. J., et al., 2017
13	Iowa	Southeast	41.1833	-91.4833	16	14	2000-2015	Poffenbarger, H. J., et al., 2017
14	Illinois	Dixon Springs	37.4347	-88.6678	13	6	2000-2012	Olson, K., et al., 2014
15	Nebraska	Mead	41.2476	-96.4695	3	21	2000-2002	Varvel, G. E., et al., 2006
16	Illinois	Urbana	40.1047	-88.2261	6	9	2000-2005	Khan, S. A., et al., 2007
17	Nebraska	Ithaca	41.0300	-96.0600	12	3	2000-2011	Jin, V. L., et al., 2015
18	Nebraska	Ithaca	41.162	-96.4115	10	6	2001-2010	Schmer, M. R., et al., 2014

[a]Selected data periods include only years after 2000. If years before 2000 were presented but the year 2000 was missing, a linear interpolation was used to estimate SOC for the year 2000.

[b]The references in this table include: Al-Kaisi, M. M., and Kwaw-Mensah, D., 2020[5]; Venterea, R. T., et al., 2006[6]; Ibrahim, M. A., et al., 2018[7]; Poffenbarger, H. J., et al., 2017[8]; Olson, K., et al., 2014[9]; Varvel, G. E., et al., 2006[10]; Khan, S. A., et al., 2007[11]; Jin, V. L., et al., 2015[12]; Schmer, M. R., et al., 2014[13].

References:

1. Kumar, M. P., Packer, B. & Koller, D. Self-paced learning for latent variable models. in *Proceedings of the 24th Annual Conference on Neural Information Processing Systems* 1189–1197 (Curran Associates, Inc., 2010).
2. Bengio, Y., Louradour, J., Collobert, R. & Weston, J. Curriculum learning. in *Proceedings of the 26th Annual International Conference on Machine Learning* (ACM, 2009). doi:10.1145/1553374.1553380.
3. Zhou, W. *et al.* Quantifying carbon budget, crop yields and their responses to environmental variability using the ecosys model for U.S. Midwestern agroecosystems. *Agric. For. Meteorol.* **307**, 108521 (2021).
4. Zhou, W. *et al.* How does uncertainty of soil organic carbon stock affect the calculation of carbon budgets and soil carbon credits for croplands in the U.S. Midwest? *Geoderma* **429**, 116254 (2023).
5. Al-Kaisi, M. M. & Kwaw-Mensah, D. Quantifying soil carbon change in a long-term tillage and crop rotation study across Iowa landscapes. *Soil Sci. Soc. Am. J.* **84**, 182–202 (2020).
6. Venterea, R. T., Baker, J. M., Dolan, M. S. & Spokas, K. A. Carbon and nitrogen storage are greater under biennial tillage in a Minnesota corn–soybean rotation. *Soil Sci. Soc. Am. J.* **70**, 1752 (2006).
7. Ibrahim, M. A., Chua-Ona, T., Liebman, M. & Thompson, M. L. Soil Organic Carbon Storage under Biofuel Cropping Systems in a Humid, Continental Climate. *Agron. J.* **110**, 1748–1753 (2018).
8. Poffenbarger, H. J. *et al.* Maximum soil organic carbon storage in Midwest U.S. cropping

systems when crops are optimally nitrogen-fertilized. *PLoS One* **12**, e0172293 (2017).

9. Olson, K., Ebelhar, S. A. & Lang, J. M. Long-term effects of cover crops on crop yields, soil organic carbon stocks and sequestration. *Open J. Soil Sci.* **04**, 284–292 (2014).

10. Varvel, G. E. Soil organic carbon changes in diversified rotations of the western corn belt. *Soil Sci. Soc. Am. J.* **70**, 426–433 (2006).

11. Khan, S. A., Mulvaney, R. L., Ellsworth, T. R. & Boast, C. W. The myth of nitrogen fertilization for soil carbon sequestration. *J. Environ. Qual.* **36**, 1821–1832 (2007).

12. Jin, V. L. *et al.* Twelve years of Stover removal increases soil erosion potential without impacting yield. *Soil Sci. Soc. Am. J.* **79**, 1169–1178 (2015).

13. Schmer, M. R., Jin, V. L., Wienhold, B. J., Varvel, G. E. & Follett, R. F. Tillage and residue management effects on soil carbon and nitrogen under irrigated continuous corn. *Soil Sci. Soc. Am. J.* **78**, 1987–1996 (2014).
