# Supplementary material for: Knowledge-guided machine learning can improve carbon cycle quantification in agroecosystems
Source: Nat Commun. 2024 Jan 8;15:357. doi: 10.1038/s41467-023-43860-5 (PMC10774286; doi:10.1038/s41467-023-43860-5)
Supplement: Supplementary file 3 — Reporting Summary [file 41467_2023_43860_MOESM3_ESM.pdf]

nature portfolio

Corresponding author(s): Zhenong Jin; Kaiyu Guan

Last updated by author(s): Nov 18, 2023

Reporting Summary

Nature Portfolio wishes to improve the reproducibility of the work that we publish. This form provides structure for consistency and transparency in reporting. For further information on Nature Portfolio policies, see our Editorial Policies and the Editorial Policy Checklist.

Statistics

For all statistical analyses, confirm that the following items are present in the figure legend, table legend, main text, or Methods section.

n/a	Confirmed	
[ ]	[x]	The exact sample size (*n*) for each experimental group/condition, given as a discrete number and unit of measurement
[ ]	[x]	A statement on whether measurements were taken from distinct samples or whether the same sample was measured repeatedly
[x]	[ ]	The statistical test(s) used AND whether they are one- or two-sided<br>*Only common tests should be described solely by name; describe more complex techniques in the Methods section.*
[x]	[ ]	A description of all covariates tested
[x]	[ ]	A description of any assumptions or corrections, such as tests of normality and adjustment for multiple comparisons
[ ]	[x]	A full description of the statistical parameters including central tendency (e.g. means) or other basic estimates (e.g. regression coefficient) AND variation (e.g. standard deviation) or associated estimates of uncertainty (e.g. confidence intervals)
[x]	[ ]	For null hypothesis testing, the test statistic (e.g. *F*, *t*, *r*) with confidence intervals, effect sizes, degrees of freedom and *P* value noted<br>*Give P values as exact values whenever suitable.*
[x]	[ ]	For Bayesian analysis, information on the choice of priors and Markov chain Monte Carlo settings
[x]	[ ]	For hierarchical and complex designs, identification of the appropriate level for tests and full reporting of outcomes
[ ]	[x]	Estimates of effect sizes (e.g. Cohen's *d*, Pearson's *r*), indicating how they were calculated

Our web collection on statistics for biologists contains articles on many of the points above.

Software and code

Policy information about availability of computer code

Data collection	FLUXNET data collection: OneFLUX (https://github.com/fluxnet/ONEFlux)
Data analysis	Model synthetic data: Ecosys process-based model (https://github.com/jinyun1tang/ECOSYS). All other data analysis was conducted using Python (version 3.7.11). Main package: Pytorch (version 1.6.0). The source codes for data processing and an executable python library of KGML-ag-Carbon models for running demo data are accessible through Zenodo under accession code https://doi.org/10.5281/zenodo.10155516.

For manuscripts utilizing custom algorithms or software that are central to the research but not yet described in published literature, software must be made available to editors and reviewers. We strongly encourage code deposition in a community repository (e.g. GitHub). See the Nature Portfolio guidelines for submitting code & software for further information.

Data

Policy information about availability of data

All manuscripts must include a data availability statement. This statement should provide the following information, where applicable:

- Accession codes, unique identifiers, or web links for publicly available datasets
- A description of any restrictions on data availability
- For clinical datasets or third party data, please ensure that the statement adheres to our policy

All data used in this study are publicly available as detailed in the Methods. Briefly, the NLDAS-2 data used in study is available at https://ldas.gsfc.nasa.gov/nldas/nldas-2-forcing-data; gSSURGO is available at https://www.nrcs.usda.gov/resources/data-and-reports/description-of-gridded-soil-survey-geographic-gssurgo-

database; the corn and soybean yield data is available at https://quickstats.nass.usda.gov/; the CDL data is available at https://croplandcros.scinet.usda.gov/; the CSDL data is available in Zenodo under accession code https://doi.org/10.5281/zenodo.4571628; the SLOPE GPP data is available at https://cabbi.bio/cabbi-data-slope-daily-and-250-m-gross-primary-productivity-gpp-for-the-conus-2000-2019/ the benchmark TRENDY-v9 data is available at https://www.wdc-climate.de/ui/entry?acronym=DKRZ_LTA_891_ds00012; and the organic carbon density data used in this study is available in SoilGrids under accession code https://files.isric.org/soilgrids/latest/data/ocd/. The aggregated KGML-ag-Carbon predictions at 0.5 degree generated in this study are provided in Source Data file, which has been deposited in the Zenodo database under accession code https://doi.org/10.5281/zenodo.10155516.

Human research participants

Policy information about studies involving human research participants and Sex and Gender in Research.

Reporting on sex and gender	n/a
Population characteristics	n/a
Recruitment	n/a
Ethics oversight	n/a

Note that full information on the approval of the study protocol must also be provided in the manuscript.

Field-specific reporting

Please select the one below that is the best fit for your research. If you are not sure, read the appropriate sections before making your selection.

☐ Life sciences ☐ Behavioural & social sciences ☒ Ecological, evolutionary & environmental sciences

For a reference copy of the document with all sections, see nature.com/documents/nr-reporting-summary-flat.pdf

Ecological, evolutionary & environmental sciences study design

All studies must disclose on these points even when the disclosure is negative.

Study description	We outlined a systematic solution for developing/training/validating the knowledge-guided machine learning (KGML) model for carbon budget quantification in the agroecosystem. The developed KGML's performance was investigated over the US Midwest region, benchmarking with existing conventional approaches, from multiple aspects including accuracy, data use efficiency, out-of-sample scenario generalizability and interpretability.
Research sample	The study used a diverse dataset for agroecosystem carbon budget quantification, including process-based model outputs (10,335 samples from 293 counties), flux tower data (FLUXNET and Ameriflux, total 11 sites), USDA yield data, remotely sensed GPP, and soil and climate information, covering 637 counties in the US Midwest. This sample represents corn/soybean rotation systems, a dominant agroecosystem in this region, highlighting our focus on large-scale, real-world agricultural practices and their carbon budget implications.
Sampling strategy	The sampling strategy was comprehensive, focusing on the US Midwest from 2000 to 2020. The selection of this region and time frame was based on its significance in global crop production and carbon cycling. Datasets were collected from public and remote sensing sources, ensuring coverage and relevance. The synthetic data were generated for 293 counties within this region to enrich the sample and enhance the model's predictive capability.
Data collection	Data collection involved downloading datasets from online sources, ensuring a broad and representative scope. This approach allowed for integrating various data types, including climate, soil, crop type, and carbon budget information, into the KGML framework.
Timing and spatial scale	The NASS Yield data are at county spatial scale and annual timing scale, during 2000-2020, at 637 counties. The flux tower observations have various length of data (randing from 4 to 19 years data) within the period of 2000-2020, at daily timing scale. Remotely sensed GPP is at daily timing scale and 250m spatial scale, during 2000-2020, over the US Midwest. Crop type is converted to the same spatial scale as GPP but with an annual timing scale. gSSURGO is converted to the same spatial scale as GPP but is constant over time. NLDAS-2 Climate data is at 1/8th degree spatial scale and is converted to daily time scale from hourly scale. Ecosys model generated synthetic data is at daily time scale for 10,335 locations within 293 counties. These scales were chosen to provide a detailed understanding of temporal dynamics and spatial heterogeneity in carbon fluxes across the US Midwest.
Data exclusions	Data exclusion criteria were primarily based on the completeness of necessary information. Samples lacking essential climate, soil, crop, or carbon budget data were omitted to maintain the integrity and reliability of the analysis.
Reproducibility	Ensemble experiments were conducted to test the reproducibility of each experiments.
Randomization	During training, the samples were random selected to be in training/validation/testing data sets. For robustness test, samples were allocated into different groups based on their spatial location (e.g. sites within the same county will be grouped) for training/validation/testing, and cross-validation were conducted to test the performance in each group. Samples were also allocated based on

	time into three groups during robust test for training/validation/testing, by random sampling two years multiple times. Moreover, the effects of sample sizes were detected by using different sizes of samples to train/validate the model and testing the model with an independent dataset.
Blinding	Blinding was not applicable in our study as it is a model development study on carbon budget quantification.

Did the study involve field work? ☐ Yes ☒ No

Reporting for specific materials, systems and methods

We require information from authors about some types of materials, experimental systems and methods used in many studies. Here, indicate whether each material, system or method listed is relevant to your study. If you are not sure if a list item applies to your research, read the appropriate section before selecting a response.

Materials & experimental systems

n/a	Involved in the study
☒	☐ Antibodies
☒	☐ Eukaryotic cell lines
☒	☐ Palaeontology and archaeology
☒	☐ Animals and other organisms
☒	☐ Clinical data
☒	☐ Dual use research of concern

Methods

n/a	Involved in the study
☒	☐ ChIP-seq
☒	☐ Flow cytometry
☒	☐ MRI-based neuroimaging
